# Using the polio programme to deliver primary health care in Nigeria: implementation research

**DOI:** 10.2471/BLT.18.211565

**Published:** 2018-11-06

**Authors:** Samuel Bawa, Christine McNab, Loveday Nkwogu, Fiona Braka, Esther Obinya, Michael Galway, Andrew J Mirelman, Kulchumi Isa Hammanyero, Garba Safiyanu, Martin Chukwuji, Kennedy Ongwae, Pascal Mkanda, Melissa Corkum, Lea Hegg, Deanna Tollefson, Sani Umar, Sunday Audu, Hassan Gunda, Modu Chinta, Anne Eudes Jean Baptiste, Murtala Bagana, Faisal Shuaib

**Affiliations:** a World Health Organization, Nigeria Country Office, UN House, 617/618 Diplomatic Drive, Central Area District, Abuja. 900001, Nigeria.; b Independent Public Health Consultant, Toronto, Canada.; c United Nations Children’s Fund, Nigeria Country Office, Abuja, Nigeria.; d Bill & Melinda Gates Foundation, Seattle, United States of America.; e Centre for Health Economics, University of York, York, England.; f United Nations Children’s Fund, Islamabad, Pakistan.; g World Health Organization, Regional Office for Africa, Brazzaville, Congo.; h United Nations Children’s Fund, Kabul, Afghanistan.; i World Health Organization, Kano Office, Kano, Nigeria.; j World Health Organization, Borno Office, Maiduguri, Nigeria.; k World Health Organization, Bauchi Office, Bauchi, Nigeria.; l World Health Organization, Yobe Office, Damaturu, Nigeria.; m National Primary Health Care Development Agency, Ministry of Health, Abuja, Nigeria.; Correspondence to Samuel Bawa (email: bawasa@who.int).

## Abstract

**Objective:**

To evaluate a project that integrated essential primary health-care services into the oral polio vaccine programme in hard-to-reach, underserved communities in northern Nigeria.

**Methods:**

In 2013, Nigeria’s polio emergency operation centre adopted a new approach to rapidly raise polio immunity and reduce newborn, child and maternal morbidity and mortality. We identified, trained and equipped eighty-four mobile health teams to provide free vaccination and primary-care services in 3176 hard-to-reach settlements. We conducted cross-sectional surveys of women of childbearing age in households with children younger than 5 years, in 317 randomly selected settlements, pre- and post-intervention (March 2014 and November 2015, respectively).

**Findings:**

From June 2014 to September 2015 mobile health teams delivered 2 979 408 doses of oral polio vaccine and dewormed 1 562 640 children younger than 5 years old; performed 676 678 antenatal consultations and treated 1 682 671 illnesses in women and children, including pneumonia, diarrhoea and malaria. The baseline survey found that 758 (19.6%) of 3872 children younger than 5 years had routine immunization cards and 690/3872 (17.8%) were fully immunized for their age. The endline survey found 1757/3575 children (49.1%) with routine immunization cards and 1750 (49.0%) fully immunized. Children vaccinated with 3 or more doses of oral polio vaccine increased from 2133 (55.1%) to 2666 (74.6%). Households’ use of mobile health services in the previous 6 months increased from 509/1472 (34.6%) to 2060/2426(84.9%).

**Conclusion:**

Integrating routine primary-care services into polio eradication activities in Nigeria resulted in increased coverage for supplemental oral polio vaccine doses and essential maternal, newborn and child health interventions.

## Résumé

**Objectif:**

Évaluer un projet qui intégrait des services de soins de santé primaires essentiels au programme d'administration du vaccin antipoliomyélitique oral au sein de communautés difficiles à atteindre et mal desservies au nord du Nigéria.

**Méthodes:**

En 2013, le centre d'intervention d'urgence pour la lutte contre la poliomyélite du Nigéria a adopté une nouvelle approche visant à augmenter rapidement l'immunité contre la poliomyélite et à réduire la morbidité et la mortalité néonatales, infantiles et maternelles. Nous avons sélectionné, formé et équipé quatre-vingt-quatre équipes sanitaires mobiles afin d'offrir des services de vaccination et de soins primaires gratuits dans 3176 zones de peuplement difficiles à atteindre. Nous avons mené des enquêtes transversales avant et après l'intervention (en mars 2014 et novembre 2015, respectivement) auprès de femmes en âge de procréer dans des familles comptant des enfants âgés de moins de 5 ans, au sein de 317 zones de peuplement sélectionnées aléatoirement.

**Résultats:**

Entre juin 2014 et septembre 2015, les équipes sanitaires mobiles ont fourni 2 979 408 doses de vaccins antipoliomyélitiques oraux et déparasité 1 562 640 enfants âgés de moins de 5 ans; elles ont tenu 676 678 consultations anténatales et soigné 1 682 671 maladies affectant des femmes et des enfants, parmi lesquelles la pneumonie, la diarrhée et le paludisme. L'enquête initiale a révélé que sur 3872 enfants âgés de moins de 5 ans, 758 (19,6%) avaient des fiches de vaccination systématique et 690 (17,8%) avaient reçu toutes les vaccinations correspondant à leur âge. L'enquête finale a révélé que 1757 enfants sur 3575 (49,1%) avaient des fiches de vaccination systématique et que 1750 (49,0%) avaient reçu toutes les vaccinations correspondant à leur âge. Le nombre d'enfants ayant reçu 3 doses ou plus de vaccins antipoliomyélitiques oraux est passé de 2133 (55,1%) à 2666 (74,6%). Le nombre d'utilisations par les familles des services de santé mobiles au cours des 6 mois précédents est passé de 509 sur 1472 (34,6%) à 2060 sur 2426 (84,9%).

**Conclusion:**

L'intégration des services de soins primaires systématiques dans les activités d'éradication de la poliomyélite au Nigéria a entraîné une augmentation du nombre de bénéficiaires de doses supplémentaires de vaccins antipoliomyélitiques oraux et des interventions essentielles en santé maternelle, néonatale et infantile.****

## Resumen

**Objetivo:**

Evaluar un proyecto que integró los servicios esenciales de atención primaria en el programa de vacunación oral contra la polio en comunidades de difícil acceso y desatendidas del norte de Nigeria.

**Métodos:**

En 2013, el centro de operaciones de emergencia contra la polio de Nigeria adoptó un nuevo enfoque para aumentar rápidamente la inmunidad a la enfermedad y reducir la morbilidad y la mortalidad de recién nacidos, niños y madres. Se identificaron, formaron y equiparon ochenta y cuatro equipos sanitarios móviles para proporcionar servicios gratuitos de vacunación y atención primaria en 3176 asentamientos de difícil acceso. Se realizaron encuestas transversales de mujeres en edad fértil en hogares con niños menores de 5 años, en 317 asentamientos seleccionados al azar, antes y después de la intervención (marzo de 2014 y noviembre de 2015, respectivamente).

**Resultados:**

Entre junio de 2014 y septiembre de 2015, los equipos sanitarios móviles administraron 2 979 408 dosis de la vacuna oral contra la polio y desparasitaron a 1 562 640 niños menores de 5 años, realizaron 676 678 consultas prenatales y trataron 1 682 671 enfermedades de mujeres y niños, incluidas la neumonía, la diarrea y el paludismo. La encuesta de referencia concluyó que 758 (19,6 %) de 3872 niños menores de 5 años tenían tarjetas de vacunación de rutina y 690/3872 (17,8 %) estaban totalmente inmunizados para su edad. La encuesta final reveló 1757/3575 niños (49,1 %) con tarjetas de vacunación de rutina y 1750 (49,0 %) totalmente inmunizados. Los niños vacunados con tres o más dosis de la vacuna oral contra la polio aumentaron de 2133 (55,1 %) a 2666 (74,6 %). El uso de los servicios sanitarios móviles por parte de los hogares en los seis meses anteriores aumentó de 509/1472 (34,6 %) a 2060/2426 (84,9 %).

**Conclusión:**

La integración de los servicios de atención primaria de rutina en las actividades de erradicación de la polio en Nigeria dio lugar a un aumento de la cobertura de las dosis suplementarias de la vacuna oral contra la polio y de las intervenciones esenciales de salud materna, neonatal e infantil.

## ملخص

**الغرض:**

تقييم مشروع يقوم بدمج خدمات الرعاية الصحية الأولية الضرورية في برنامج لقاح فموي لشلل الأطفال في المجتمعات التي يصعب الوصول إليها والتي تفتقر إلى الخدمات في شمال نيجيريا.

**الطريقة:**

في عام 2013، اتبع مركز عمليات الطوارئ في مجال شلل الأطفال في نيجيريا نهجاً جديداً لرفع المناعة ضد شلل الأطفال بشكل سريع سريع، وخفض المراضة والوفيات بين المواليد والأطفال والأمهات. قمنا بتحديد أربعة وأربعين فريقاً صحياً متنقلاً، وتدريبهم وتجهيزهم، وتوفير خدمات التلقيح والرعاية الأولية مجاناً في 3176 مستوطنة يصعب الوصول إليها. أجرينا مسوح مقطعية على النساء في سن الإنجاب في الأسر التي لديها أطفال تقل أعمارهم عن 5 سنوات، في 317 مستوطنة تم اختيارها عشوائياً، ما قبل وما بعد التدخل (مارس/آذار 2014 ونوفمبر/تشرين ثاني 2015، على التوالي).

**النتائج:**

من يونيو/حزيران 2014 إلى سبتمبر/أيلول 2015، قدمت الفرق الصحية المتنقلة 2979408 جرعة من اللقاح الفموي المضاد لشلل الأطفال، وساعدت في تخليص 1562640 طفلاً أصغر من 5 سنوات من الديدان، وأجرت 676678 استشارة سابقة للولادة، وعالجت 1682671 حالة مرضية في النساء والأطفال، بما في ذلك الالتهاب الرئوي والإسهال والملاريا. وجد المسح الأساسي أن 758 (19.6٪) من 3872 طفلاً أصغر من 5 سنوات كان لديهم بطاقات تحصين روتينية، وتم تحصين 690/3872 (17.8٪) بالكامل طوال أعمارهم. وجد المسح النهائي 1757/3575 طفلاً (49.1٪) لديهم بطاقات تحصين روتينية، و1750 (49.0٪) محصنين بالكامل. ازداد عدد الأطفال الذين تم تطعيمهم باستخدام 3 أو أكثر من جرعات لقاح شلل الأطفال الفموي، من 2133 (55.1٪) إلى 2666 (74.6٪). زاد استخدام الأسر للخدمات الصحية المتنقلة في الأشهر الستة السابقة من 509/1472 (34.6٪) إلى 2060/2426 (84.9٪).

**الاستنتاج:**

أدى دمج خدمات الرعاية الأولية الروتينية في أنشطة استئصال شلل الأطفال في نيجيريا، إلى تغطية متزايدة للجرعات الإضافية من اللقاح الفموي المضاد لشلل الأطفال، والتدخلات الصحية الضرورية للأمهات والمواليد والأطفال. ****

## 摘要

**目的:**

旨在评估一项在尼日尼亚北部难以到达、缺乏服务的社区开展的，将基本的初级卫生保健服务纳入口服脊髓灰质炎疫苗计划中的项目。

**方法:**

2013 年，尼日利亚小儿麻痹症急诊手术中心采用了一种新方法，以迅速提高脊髓灰质炎免疫力，从而降低新生儿、儿童和孕产妇的发病率和死亡率。我们确定，84 个接受过培训、配备装备的流动医疗队，在 3176 个难以到达的定居点提供免费疫苗接种和初级卫生保健服务。干预前和干预后（分别为 2014 年 3 月和 2015 年 11 月），我们随机选择 317 个定居点，并对在家中抚养 5 岁以下儿童的育龄妇女进行了横断面调查。

**结果:**

从 2014 年 6 月至 2015 年 9 月，流动医疗队为其接种 2979408 剂口服脊髓灰质炎疫苗，并为 1562640 名 5 岁以下儿童驱虫；进行了 676678 次产前咨询，治疗了 1682671 例妇女和儿童的病例，包括肺炎、腹泻和疟疾。基线调查发现 3872 名年龄小于 5 岁的儿童中有 758 名（占 19.6％）拥有常规免疫接种卡，3872 名儿童中的 690 名（占 17.8％）完全免疫。底线调查发现 3575 名儿童中的 1757 名 （占 49.1％）拥有常规免疫接种卡，其中 1750 名（占 49.0％）完全免疫。接种 3 剂口服脊髓灰质炎疫苗或更多剂量的儿童人数从 2133 例（占 55.1％）增至 2666 例（占 74.6％）。过去 6 个月，使用移动医疗服务的家庭从 509 户（占 1472 户的 34.6％）增至 2060 户（占 2426 户的 84.9％）。

**结论:**

将常规初级卫生保健服务纳入尼日利亚的脊髓灰质炎根除活动，可以增加补充口服脊髓灰质炎疫苗剂的覆盖率，增加基本孕产妇、新生儿和儿童卫生干预措施的覆盖率。****

## Резюме

**Цель:**

Оценка проекта по включению основной первичной медицинской помощи в программу распространения пероральной полиомиелитной вакцины для труднодоступных и плохо охваченных обслуживанием общин на севере Нигерии.

**Методы:**

В 2013 г. Нигерийский центр неотложной помощи больным полиомиелитом внедрил новый подход в отношении быстрого повышения уровня иммунитета к полиомиелиту и сокращения заболеваемости и смертности среди новорожденных, матерей и детей. Авторы выявили, обучили и оснастили восемьдесят четыре мобильные бригады медиков для бесплатной вакцинации и оказания первичных медицинских услуг в 3176 труднодоступных поселениях. В 317 поселениях, выбранных случайным образом, до и после мероприятий (в марте 2014 года и в ноябре 2015 года соответственно) были проведены перекрестные обследования женщин детородного возраста в семьях с детьми младше 5 лет.

**Результаты:**

С июня 2014 года по сентябрь 2015 года мобильные бригады медиков предоставили населению 2 979 408 доз пероральной вакцины от полиомиелита и провели глистогонную терапию 1 562 640 детям младше 5 лет; они также провели 676 678 дородовых консультаций и вылечили 1 682 671 заболевание у женщин и детей, включая пневмонию, диарею и малярию. Исходный опрос выявил, что у 758 детей младше 5 лет (19,6%) из 3872 были карточки регулярных прививок и 690 из 3872 (17,8%) были полностью привиты соответственно возрасту. В итоговом обследовании выяснилось, что 1757 из 3575 детей (49,1%) имели карточки регулярных прививок и 1750 детей (49%) были полностью привиты. Количество детей, получивших 3 и более дозы пероральной вакцины от полиомиелита, возросло с 2133 (55,1%) до 2666 (74,6%). Количество семей, использовавших услуги мобильных медицинских бригад за последние 6 месяцев возросло с 509 из 1472 (34,6%) до 2060 из 2426 (84,89%).

**Вывод:**

Объединение услуг первичной медицинской помощи с усилиями по искоренению полиомиелита в Нигерии привело к увеличению охвата дополнительными дозами поддерживающей пероральной вакцины и важными мероприятиями по охране здоровья детей, матерей и новорожденных.****

## Introduction

The World Health Organization (WHO) has called for universal health coverage (UHC) to be available for all people and communities, emphasizing the need for promotive, preventive, curative, rehabilitative and palliative health services that do not expose the user to financial hardship.^1^ Achieving UHC, including quality essential service coverage and financial protection for all, is a target of sustainable development goal (SDG) 3 to ensure healthy lives and promote well-being for all at all ages.^2^ Furthermore, the Global Vaccine Action Plan also seeks to realize a world in which all individuals and communities enjoy the full benefit of immunization, including use of immunization systems for delivery of other primary health-care programmes.^3^ Given their global reach (98.8 of 116.2 million infants receiving three doses of diphtheria–tetanus–pertussis vaccine), immunization programmes provide a platform on which to strengthen UHC.^4^ Notably, Nigeria’s national polio eradication programme’s emphasis on reaching every household is an opportunity to reduce inequities in health by reaching the most vulnerable groups of the population.^5^

According to the milestones of the Polio Eradication and Endgame Strategic Plan 2013–2018, poliovirus transmission was to be stopped globally by the end of 2014.^6^ In 2013, Nigeria was one of three remaining polio-endemic countries worldwide.^7^ However, challenges remained in achieving adequate polio vaccine coverage in Nigeria, putting the polio eradication goal at risk. The country reported 53 people with poliovirus that year, 46 (86.8%) in the northern states of Bauchi, Borno, Kano and Yobe, and five people with vaccine-derived poliovirus isolated from Borno and Adamawa states.^8^ Most were children from poor, rural families living in areas deemed hard-to-reach due to distance or geography.

People in hard-to reach settlements generally experience low coverage of basic public health services including routine immunization and maternal, newborn and child health services. In 2013, Nigeria had made progress in maternal and child health, but continued to record high estimates of newborn, under-five and maternal mortality.^9^ The country also had the 10th highest under-five mortality rate globally and the 15th highest maternal mortality of 560 per 100 000 live births (contributing to 14% of global maternal deaths, with 40 000 estimated deaths).^10^ For each of the indicators, the rates in the northern zones where polio transmission continued were as much as twice as high or more than the national figures.^11^

Nigeria had been implementing traditional polio eradication strategies, including increasing immunity through routine immunization, regularly scheduled house-to-house oral polio vaccine supplemental immunization activities and sensitive surveillance for acute flaccid paralysis. Additionally, through the polio emergency operations centre, the polio programme had been continuously innovating to improve vaccine coverage (e.g. through use of satellite mapping and vaccine carrier trackers to identify unreached areas).^12^^,^^13^ Monitoring data showed that these efforts were achieving good results and that campaigns were reaching more children with every vaccination round. However, there were still a high number of unvaccinated children, especially in underserved communities and hard-to-reach areas, demonstrated by monitoring data and the presence of polio cases.^14^ In late 2013, to address this problem, the polio emergency operations centre adopted a new approach with technical and financial support from its partners: the WHO, the United Nations Children’s Fund (UNICEF) and the Bill & Melinda Gates Foundation. The aim was to reach more children with routine immunizations, including oral polio vaccine, while also providing maternal and child health survival interventions during mobile outreach sessions in six priority northern states of Nigeria. This integrated approach became known as the Hard to Reach communities project. The project aimed to raise population immunity to polio and enable hard-to-reach and vulnerable communities to access essential primary health-care services including maternal, newborn and child health care.

This manuscript presents the evaluation of the project. We aimed to measure the project’s effectiveness by assessing changes in immunization coverage, basic public health knowledge and access to and use of public health services in the selected communities.

## Methods

### Project implementation

We implemented the project for 18 months between 1 June 2014 and 30 September 2015. The project proposed to expand on an existing mobile outreach strategy for routine immunization that was part of Nigeria’s national policy but not consistently implemented. The enhanced strategy provided routine immunization together with a basic integrated package of primary health-care interventions focused on maternal, newborn and child health.

The emergency operations centre selected a total of 3176 settlements in six northern states (Bauchi, Borno, Kaduna, Kano, Katsina and Yobe). Hard-to-reach settlements were communities that had geographically difficult terrain with any local or state border, scattered households, nomadic populations, waterlogged or riverine areas, or where it was difficult to access the health-care facilities due to insecurity. UNICEF managed implementation in Kaduna and Katsina, and WHO managed implementation in Bauchi, Borno, Kano and Yobe.

Each of the 84 mobile health teams comprised at least one nurse or midwife, a community health extension worker and a health records assistant. Staff were identified, trained and equipped with weighing scales, stethoscope, health commodities (e.g. essential drugs and consumables as contained in the UNICEF Emergency Health Kit) and recording tools. The 3-day training was provided within each state by facilitators using materials adapted from the Integrated Management of Childhood Illnesses and the Maternal, Neonatal and Child Health Week modules, with opportunities for refresher sessions during regular, monthly review meetings. Each team was assigned a specific number of settlements. Teams conducted mobile outreach visits to three to four settlements each week, and were expected to visit their assigned settlements once every 3 months. Their salaries were paid directly by UNICEF and WHO under non-staff consultancy contracts.

The teams coordinated closely with local health-care personnel and the community. They worked directly with the routine immunization focal person of the health facility in the settlement catchment area. Volunteer community mobilizers, usually women from the settlements, were engaged and paid a small stipend to announce the outreach dates and promote basic public health behaviours. These volunteers were trained in their respective wards of residence on community engagement and defaulter tracking. 

The project also provided funds for transportation to the teams depending on route conditions (e.g. to hire four-wheel drive vehicle, motorcycle or boat). Team movements were monitored by local government facilitators, using checklists and mobile devices (a geographical information tracking system), which showed real-time movements for the purposes of monitoring settlement coverage and team security. Supervisors from partner organizations and the government project focal persons made supervisory field visits. There was an established programme review through monthly and quarterly review meetings at the state and subnational levels, respectively.

We summarized the records generated during each outreach session (numbers of children vaccinated, vitamin A provided, children dewormed and nutritional screenings done; numbers of people seen and treated for ailments) and sent them via mobile devices to a server domiciled with an independent geographical information system provider. Weekly summaries were collated to monitor the sessions conducted and coverage of services; and transmitted to the local and state government levels.

During the mobile outreach sessions, women and children in hard-to-reach settlements received a range of integrated health services. For example, pregnant women received antenatal care, malaria preventive therapy, iron folate, tetanus toxoid vaccine and treatment of illnesses (e.g. malaria and respiratory infections) or referral for care. Children aged 0–59 months received a full complement of routine immunizations (including oral polio vaccine), vitamin A supplements, deworming, diagnosis and referral for malnutrition, treatment of diarrhoea, pneumonia and malaria and additional referrals as required. In addition, all women attending outreach session were provided with health education on key household practices (hand washing, personal hygiene and infant feeding including exclusive breastfeeding).

### Study design

To assess changes in coverage for polio immunization and maternal, newborn and child health services, we conducted cross-sectional surveys at the start (baseline, March 2014) and after the implementation of the project (endline, November 2015). We used a simple random sampling method to select 317 (10%) of the 3176 hard-to-reach settlements where the project was implemented.

### Data collection

A cross-sectional survey was made of women of childbearing age (15–49 years) in households containing at least one child aged 0–59 months (10 households in each settlement). In selected settlements with 10 or less households, all the households in the settlement were sampled and if 10 eligible mothers were not obtained, the surveyor moves to the nearest settlement within the same local government area and completed the process. In selected settlements with more than 10 households, the surveyor randomly selected the first household to be sampled and continued in a systematic way until 10 eligible mothers were obtained. 

A total of 206 independent, trained surveyors administer the standardized questionnaires. The questionnaire asked about the women’s demographic characteristics; knowledge of common preventable diseases; household’s access to services and coverage; and household member’s use of the mobile health sessions in their communities. The women were also asked about vaccinations for children younger than 5 years old in the household. Interviewers asked to see the vaccination card and records of polio vaccinations, asked the reason why any child had not been vaccinated and verified children’s tuberculosis vaccine scars. Surveys were administered over a period of 7–10 days at baseline (15–24 March 2014) and endline (3–16 October 2015). Due to population dynamics, for example, nomadic populations and displacement due to insecurity, the survey participants were not the same at baseline and endline. Households and respondents were not included in our second survey if they had not lived in the community for more than 6 months. Similarly, the settlements were not always the same, but must have been in the sampling frame, i.e. the selected settlements where the intervention was implemented.

We also collected data from the project records on the services provided during the mobile outreaches session, which included summaries of children vaccinated, numbers of clients seen and the diseases treated.

### Data analyses

Analysis for the baseline and endline surveys were conducted separately to determine outcomes and to evaluate the integration of services. Analyses included comparisons of reported data across the six states during the studied periods. Descriptive analyses were used to compare information across the selected variables.

### Ethical considerations

The surveys formed part of the monitoring and evaluation activities of the Hard-to-Reach communities project, that was not intended as research work, but instead as an intervention to improve vaccination uptake among hard-to-reach communities. However, the government of Nigeria approved the project as part of the Global Polio Eradication Initiative activities to achieve the goals of the national polio eradication emergency plans and granted permission for the activities in the project.

We obtained ethical clearance from the Bauchi state health research ethics committee. The survey assistants obtained informed consent from each survey participant after interpreting and explaining the consent section of the questionnaire in the participant’s local language. Those who gave their consent continued with the interview.

## Results

### Project outcomes 

During the project period, the mobile outreach sessions delivered 2 979 408 supplemental doses of oral polio vaccine to children younger than 5 years and 346 880 children were fully immunized (measles vaccine was used as a proxy for full immunization). More than 1.5 million children were dewormed; 676 678 antenatal care consultations were performed; 1 359 323 women were provided information on exclusive breastfeeding and more than 1.68 million illnesses among women and children were treated, including pneumonia, diarrhoea and malaria (Table 1).

**Table 1:** Results of programme interventions in six states in the hard-to-reach communities project in Nigeria, June 2014 to September 2016

Variable	State						
	Kaduna	Katsina	Bauchi	Borno	Kano	Yobe	Total
**Population of target communities^a^**	8 152 952	7 784 740	6 533 157	5 799 337	12 983 043	3 274 833	44 528 062
**Key interventions delivered**							
Total no. of doses of oral polio vaccine given	338 910	577 317	641 107	598 454	370 342	453 278	2 979 408
No. of children (aged 0–11 months) fully oral polio vaccine immunized	13 303	27 449	57 612	62 758	31 712	33 049	225 883
No. of children fully immunized (measles vaccination)	22 392	61 042	93 465	83 765	20 917	65 299	346 880
No. of children receiving growth monitoring	309 731	388 175	378 633	211 320	270 673	282 622	1 841 154
No. of children given vitamin A	204 987	371 362	359 743	209 850	220 256	288 786	1 654 984
No. of children dewormed	210 502	346 617	361 161	201 471	197 901	244 988	1 562 640
No. of women reached with message on exclusive breastfeeding	220 609	251 830	258 686	140 121	290 819	197 258	1 359 323
No. of adults reached with education on key household practices^b^ and health promotion	236 799	385 656	293 861	164 018	315 849	210 345	1 606 528
No. of minor ailments treated	151 346	263 707	225 234	348 312	326 116	367 956	1 682 671
No. of antenatal consultations done	27 312	55 795	239 077	127 569	55 186	171 739	676 678
No. of tetanus toxoid vaccine doses given	42 458	57 156	130 354	129 980	36 823	91 128	487 899
No. of iron folate doses given	38 766	57 452	220 548	91 051	42 144	71 319	521 280
No. of malaria intermittent preventive treatment doses given	24 038	50 605	67 425	80 931	29 729	37 718	290 446

^a^ Projected population by states from Nigeria 2006 population census.

^b^ Key household practices of integrated management of childhood illnesses.

### Demographic characteristics

At baseline, we interviewed 3166 women with 3873 children younger than 5 years old. The endline survey included interviews with 2426 women with 4651 children younger than 5 years old.

Table 2 presents the demographic characteristics of the women. In the baseline sample, Fulani were the major ethnic group (1077; 37.1 %) and one-quarter of women (730; 25.1%) were from nomadic populations. At baseline, many women (1216; 41.8%) had no education and 1382 (47.6%) had Koranic rote learning only. Most of the women were crop farmers (1112; 38.3%). In the endline sample, the distribution of occupational characteristics was similar, but there was a lower proportion of women with no education (912; 37.6%) and Hausa were the majority ethnic group (987; 40.7%). 

**Table 2:** Demographic profile of household caregivers surveyed in the hard-to-reach communities project in Nigeria, at baseline (March 2014) and endline (November 2015)

Category	No. (%) of respondents	
	Baseline *n* = 2906	Endline *n* = 2426
**Ethnic group**		
Fulani	1077 (37.1)	931 (38.4)
Hausa	981 (33.8)	987 (40.7)
Kanuri	540 (18.6)	172 (7.1)
Shuwa or Arab	34 (1.2)	2 (0.1)
Margi	47 (1.6)	0 (0.0)
Other	227 (7.8)	334 (13.8)
**Residential status**		
Nomadic	730 (25.1)	769 (31.7)
Settled	2126 (73.2)	1657 (68.3)
No response	50 (1.7)	0 (0.0)
**Educational level**		
None	1216 (41.8)	912 (37.6)
Koranic	1382 (47.6)	1312 (54.1)
Primary	143 (4.9)	158 (6.5)
Secondary	95 (3.3)	42 (1.7)
Post-secondary	13 (0.4)	2 (0.1)
No response	57 (2.0)	0 (0.0)
**Occupation**		
Home keeper	461 (15.9)	467 (19.2)
Animal product seller	600 (20.6)	511 (21.1)
Casual labourer	96 (3.3)	171 (7.0)
Civil servant	31 (1.1)	26 (1.1)
Crop farmer	1112 (38.3)	715 (29.5)
Trader	283 (9.7)	482 (19.9)
Other	304 (10.5)	54 (2.2)
No response	19 (0.7)	0 (0.0)

Note: Inconsistencies arise in some values due to rounding.

### Awareness of diseases

At endline there was higher awareness about vaccine-preventable diseases and use of mobile outreach services among household caregivers. In the baseline survey, of the 2204 (75.8%) women aware of vaccine-preventable diseases, 523 (23.7%) were aware of measles and 484 (22.0%) of polio. In the endline survey, of 2105 (86.8%) women aware of vaccine-preventable diseases, 1806 (85.8%) and 1544 (73.3%) were aware of measles and polio, respectively. The numbers of women aware of cerebrospinal meningitis were 18 (0.8%) at baseline and 156 (7.4%) at endline. An increase in the mothers’ level of awareness was also recorded for tuberculosis, yellow fever and pertussis (Table 3).

**Table 3:** Vaccination coverage for children younger than 5 years old and household caregivers’ awareness of vaccine-preventable diseases in the hard-to-reach communities project in Nigeria, at baseline (March 2014) and endline (November 2015)

Variable	No. (%) of respondents	
	Baseline	Endline
**Children’s vaccination history**		
Total children sampled	3872 (100.0)	3575 (100.0)
Age of children sampled, months		
< 6	518 (13.4)	330 (9.2)
6–8	275 (7.1)	330 (9.2)
9–23	1259 (32.5)	1271 (35.6)
24–35	572 (14.8)	575 (16.1)
≥ 36	1210 (31.3)	1069 (29.9)
No response	38 (1.0)	0 (0.0)
Children with routine immunization card	758 (19.6)	1757 (49.1)
Children fully immunized for age at the time of survey	690 (17.8)	1750 (49.0)
Children with visible BCG scar	904 (23.3)	580 (16.2)
Children given supplemental oral polio vaccine, no. of doses		
0	445 (11.5)	167 (4.7)
1–3	1045 (27.0)	742 (20.8)
> 3	2133 (55.1)	2666 (74.6)
Don’t know	249 (6.4)	0 (0.0)
Reasons for zero dose of oral polio vaccine^a^		
Caregiver refused vaccination	152 (36.9)	23 (13.8)
**Awareness of vaccine-preventable diseases**		
Total caregivers interviewed	2906 (100.0)	2426 (100.0)
Caregiver aware of any vaccine-preventable diseases	2204 (75.8)	2105 (86.8)
Caregiver aware of specific diseases^b^		
Cerebrospinal meningitis	18 (0.8)	156 (7.4)
Tuberculosis	46 (2.1)	250 (11.9)
Yellow fever	40 (1.8)	619 (29.4)
Pertussis	101 (4.6)	843 (40.0)
Polio	484 (22.0)	1544 (73.3)
Measles	523 (23.7)	1806 (85.8)

BCG: bacille Calmette–Guérin.

^a^ The denominator for percentages is the number of children with zero doses of supplemental oral polio vaccine.

^b^ The denominator for percentages is the number of caregivers aware of any vaccine-preventable disease.

Note: Inconsistencies arise in some values due to rounding.

### Immunizations

At baseline, 758 out of 3872 children in the sample (19.6%) had routine immunization cards and 690 (17.8%) children were fully immunized for their age. At endline, 1757 of 3575 children (49.1%) had routine immunization cards and 1750 (49.0%) were fully immunized. The number of children with zero doses of polio immunization decreased from 445 (11.5%) at baseline to 167 (4.7%) at endline. The main reason for zero doses was caregivers refusing vaccination, with numbers reducing from 152 (36.9%) to 23 (13.9%; Table 3).

### Access to services

Table 4 shows that the reported provision of mobile routine immunization outreach services coordinated through the nearest health facility in the 6 months before the survey increased from 34.6% (509/1472) to 84.9% (2060/2426). There was also an increase in reported access to free health services in the 2 weeks before the survey from 8.4% (122/1447) at baseline to 75.9% (858/1130) at endline.

**Table 4:** Access to and use of health-care services reported by household caregivers in the hard-to-reach communities project in Nigeria, at baseline (March 2014) and endline (November 2015)

Variable	Baseline		Endline	
	Total respondents	No. (%) agreeing	Total respondents	No. (%) agreeing
Used mobile routine immunization service in the previous 6 months	1472	509 (34.6)	2426	2060 (84.9)
Aware of availability of free mobile health-care service	1830	396 (21.6)	2426	1975 (81.4)
Any member of the family accessed mobile health-care service in the previous 2 weeks	1447	122 (8.4)	1130	858 (75.9)

## Discussion

The integration of the polio eradication platform with additional primary health services to underserved communities demonstrates how an integration of outreach services can increase coverage and knowledge, reinforcing efforts to attain UHC and SDG 3.^1^^,^^15^^,^^16^ The model could be applied in areas where polio vaccine and routine immunization mobile outreach programmes could reach normally hard-to-reach and vulnerable communities with maternal, newborn and child health services they might not otherwise access.

The project appeared successful in increasing polio vaccination coverage as well as routine immunization and basic maternal, newborn and child health services among the selected communities, some of whom may never had had contact with the health system. There have been no polio cases or poliovirus-positive environmental samples in any of the settlements since the project began.^17^ The inclusion of volunteer community mobilizers helped to foster community involvement and demand for polio vaccine and other health-care services.^16^^,^^18^ Communities accessed free primary health care at mobile services. Knowledge about public health practices and services and some disease conditions improved. However, knowledge about pertussis and yellow fever decreased. This may not be unconnected with the fact that with the drive for polio eradication, sensitization on other disease conditions may have been down-played.

Critical to the success of the project was securing the resources needed to train, equip, transport, supervise and remunerate the workforce. Of course, the additional staffing, transport, community mobilizers and costs of maternal, newborn and child health supplies required more funds than would oral polio vaccine or routine immunization sessions alone. However, by packaging these additional services together with polio vaccines and routine immunization outreach, economies of scale may be achieved. This hypothesis would benefit from further cost–benefit and cost–effectiveness studies.^19^

Another key component was stakeholder engagement and national and state government involvement in project design, monitoring, supervision and reviews, together with community and traditional leaders. This is similar to Cambodia’s integrated immunization programme, which has planned a national level monitoring strategy aimed at provision of adequate management support to provinces and districts.^19^

A longer-term impact evaluation would be required to measure outcomes in terms of reduction in morbidity and mortality associated with the interventions offered.^20^^,^^21^

We experienced some challenges, however, which may or may not arise if such an approach were applied in other country settings. Security, for example, proved to be a major challenge particularly in Borno and Yobe states where the Boko Haram militant group insurgency caused insecurity and population displacement.^22^^,^^23^ The project adjusted by using funds to deliver integrated services to camps of internally displaced persons. Armed robbery and intercommunal clashes were also concerns. In Kaduna state, for example, the project could no longer serve one local government area due to prolonged intercommunal clashes, and UNICEF had to select another local government area in its place. A lesson is that programme implementation should be reviewed regularly so it can be adjusted flexibly, especially where health systems are affected by protracted humanitarian crises.

Health workers also observed that people from non-targeted settlements routinely arrived to seek services from the mobile teams, suggesting strong community demand. However, this also made target populations more difficult to enumerate and created challenges for stock management.

Our evaluation was not without limitations. Data collection was not uniform across the implementing states as WHO and UNICEF managed their programmes slightly differently, hindering comparative analysis across all data points. For instance, while the teams in Bauchi, Borno and Kano states used registers to capture treatment of clients, the in Kaduna and Kano teams used tally sheets. Project reviews found health workers were not uniformly aware of the package of services to be offered and at times lacked sufficient skills to deliver the full package. This was similar to other findings where coverage and other quality indicators of integrated services were not always disaggregated by the service delivery approach.^24^ Future efforts should address the need for uniformity of data collection variables for comparison and more uniformity in training materials and supervision of the mobile health teams.

Given the demanding nature of the work and the scarcity of health-care workers in some areas, it was at times difficult to identify and retain qualified staff and ensure a full complement of services per mobile team. Finally, there was the challenge of not depleting the mainstream health personnel by employing health workers seeking additional income, also reported in other studies.^19^

While the project promoted referrals (arising from severe acute malnutrition, for example) these were not always possible if there was no referral centre nearby or if fees at the health facility were prohibitively costly for clients.

Overall, the project demonstrated how the polio platform could be used to deliver an integrated mobile health strategy that helps to achieve greater equity for marginalized and vulnerable populations. Funding a dedicated, trained and equipped team of health workers to target hard-to-reach communities will have an impact in improving equitable access to basic health services. As a polio legacy project, it has also demonstrated how an integrated routine immunization mobile strategy could be planned, implemented and monitored to achieve greater equity for marginalized and vulnerable populations living in hard-to-reach areas.

As the project in some of the states improved delivery of health care to underserved areas, some state governments (Kano and Yobe) are offering continued support to sustain funding for its delivery. Furthermore, WHO’s emergency programme, with funding from Borno and Yobe states, are conducting ongoing outreach in those states. The Canadian government is funding an ongoing collaboration with UNICEF in Niger, Jigawa, Taraba and Zamfara states.

The polio eradication platform, which usually includes expertise in how to plan to reach every child younger than 5 years, including those in vulnerable communities, could be used to plan integrated delivery of primary health-care services. Furthermore, countries that include outreach sessions in their immunization strategy can integrate essential maternal, newborn and child health services and deliver them together with routine immunization. These efforts will require additional resources and continued commitment from governments. The Hard-to-Reach communities project demonstrates that those resources can result in more equitable access to health care for the most vulnerable, a key to UHC and achievement of SDG 3.
